# Supplementary material for: VarID2 quantifies gene expression noise dynamics and unveils functional heterogeneity of ageing hematopoietic stem cells
Source: Genome Biol. 2023 Jun 23;24:148. doi: 10.1186/s13059-023-02974-1 (PMC10290360; doi:10.1186/s13059-023-02974-1)
Supplement: Supplementary file 1 — Additional file 1: Supplementary figures and tables. [file 13059_2023_2974_MOESM1_ESM.docx]

**
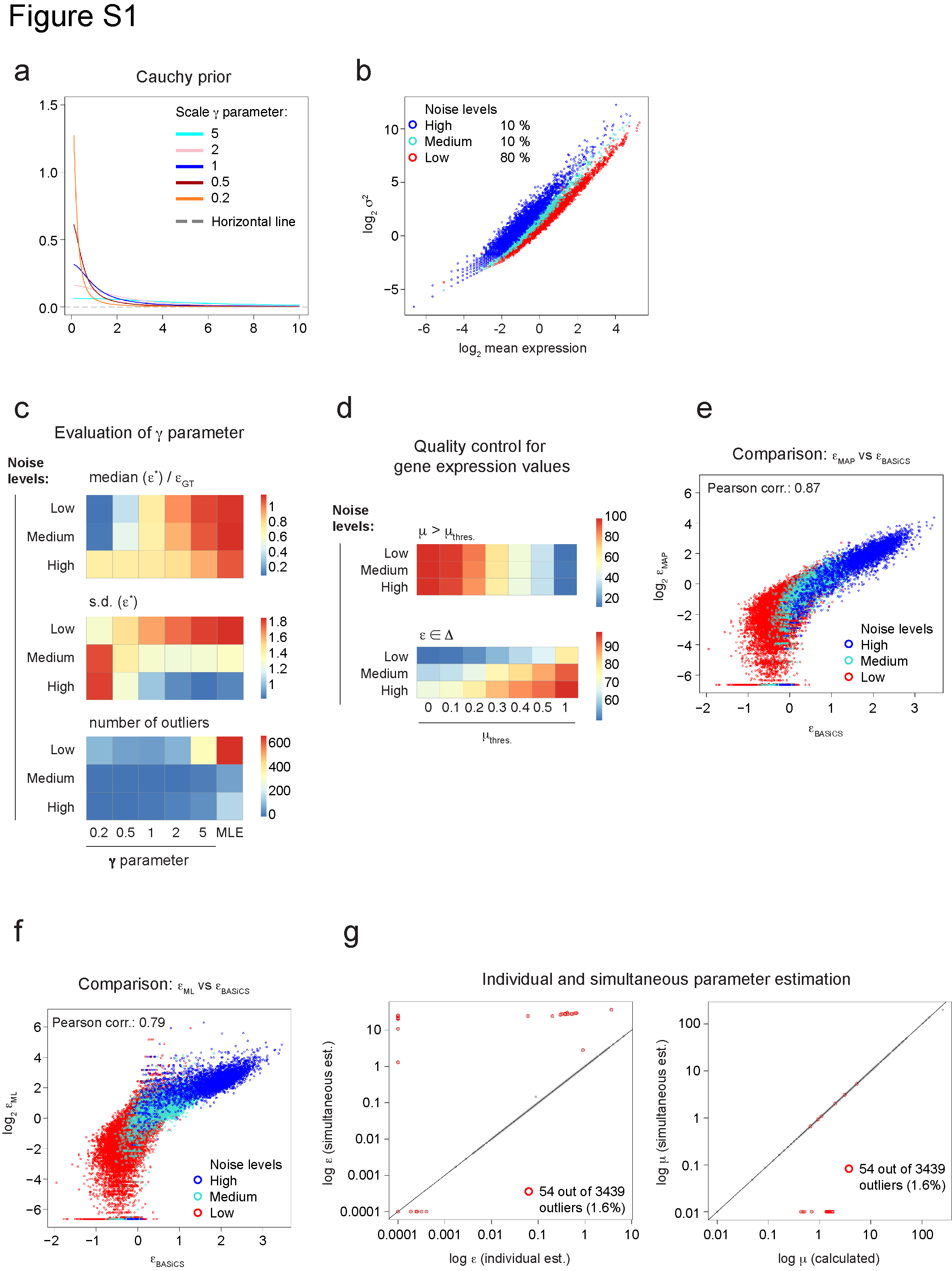
**

**Figure S1. Local decomposition of gene expression noise in cell state space.** **a** Probability density function of the Cauchy distribution with different values of the scale parameter $\gamma$. Only positive values are considered for the Cauchy distribution. Location parameter was set to zero. **b** Variance as a function of the mean on a logarithmic scale for a simulated dataset with genes grouped into three levels of biological noise (“Methods”). **c** Tests for hyperparameter $\gamma$ selection, based on the ratio: $median (\varepsilon^{*})/\varepsilon_{GT}$ (GT: Ground Truth); the standard deviation $s.d. (\varepsilon^{*})$; and number of outliers, given by $\varepsilon>\varepsilon_{GT}+s.d.(\varepsilon^{*})$. $\varepsilon^{*}$ estimates correspond to genes whose mean expression meets the condition: ${log}_{2} (\mu_{i})>1$. **d** Assessment of quality control based on gene expression thresholds. Top: percentage of high confidence genes with mean expression higher than the indicated thresholds (arranged in columns). Bottom: percentage of high confidence genes with $\varepsilon$ estimates within $\Delta$, representing the 2-fold interval around the ground truth ($\varepsilon_{GT}$). **e** Comparison of $\varepsilon_{MAP}$ estimates, with $\varepsilon_{BASiCS}$, the dispersion parameter $\varepsilon$ computed by BASiCS. **f** As (**e**), but comparison of maximum likelihood estimates $\varepsilon_{ML}$ and $\varepsilon_{BASiCS}$ estimates. **g** Left: $\varepsilon$ estimates inferred by 2D inference ($\mu$ and $\varepsilon$) versus 1D inference ($\varepsilon$ only with calculated $\mu$, individual MAP estimation, following the strategy of VarID2) for an example neighborhood (mouse Kit+ bone marrow dataset [22]). Outlier points with strongly diverging estimates are highlighted in red. Right: similar to the left plot, but showing $\mu$ estimates and the outliers selected from the left panel are highlighted in red. Outliers correspond to data points with vanishing $\mu$ estimates, whereas the calculated mean is a positive number, suggesting that 2D inference did not converge to the true value of $\mu$. A pseudocount of 10^-4^ and 10^-2^ was added to $\varepsilon$ and $\mu$ values, respectively.

**
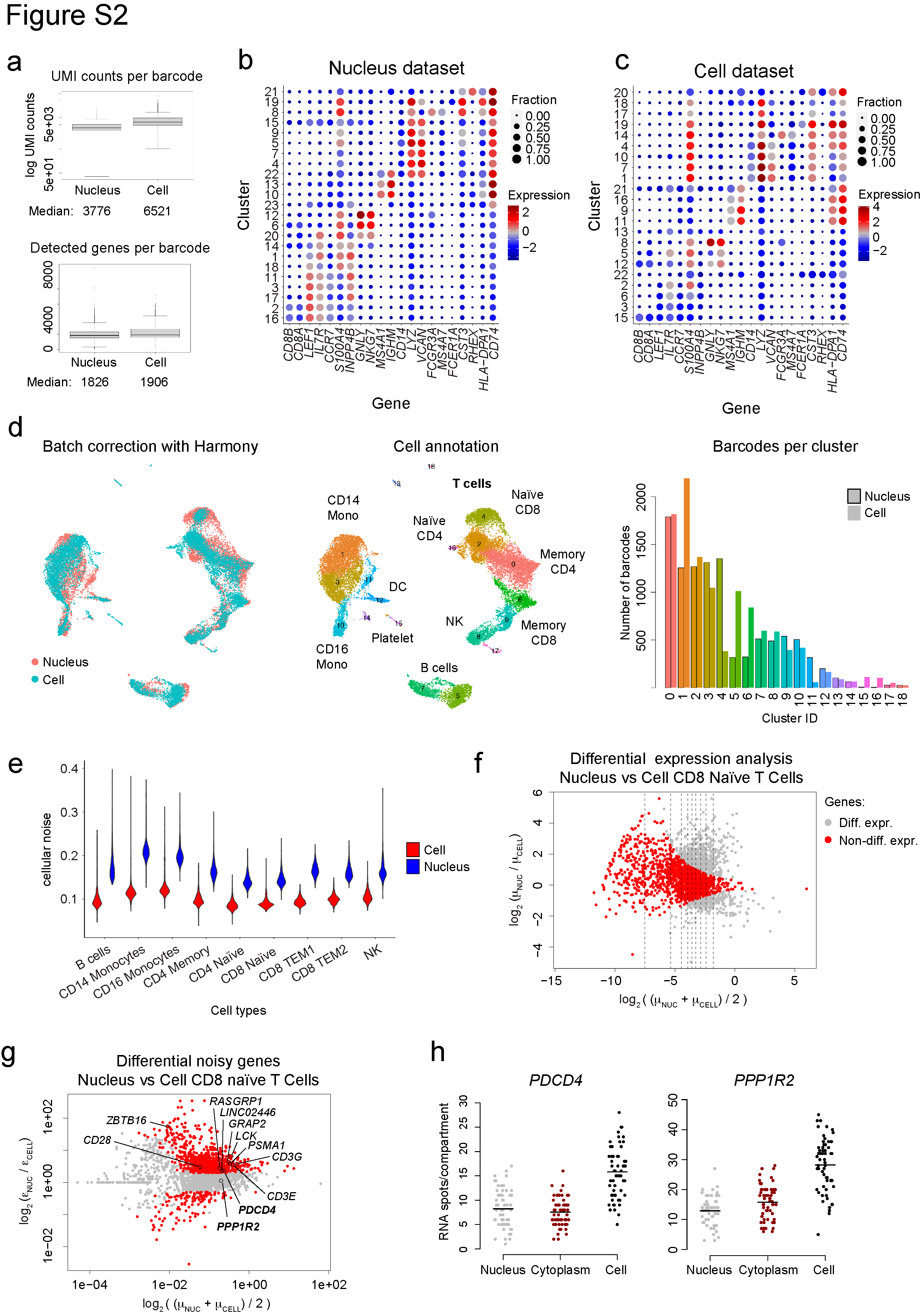
**

**Figure S2. Elevated noise levels of nuclear versus whole-cell transcriptomes in human PBMCs. a** Number of UMI counts (top) and number of detected genes per barcode (bottom) for snRNA-seq (Nucleus) and scRNA-seq (Cell) data of human PBMCs. Boxes indicate inter-quartile range (IQR), and whiskers correspond to ±1.5*IQR of the box limits. Outliers beyond the whisker limits are depicted. **b** Expression of relevant marker genes across clusters in the snRNA-seq dataset. Dot size indicates the fraction of cells with positive expression and dot color highlights logarithmic expression (log_2_) calculated across clusters. **c** As (**b**), but expression detected in the scRNA-seq dataset. **d** Batch effect correction with Harmony. UMAP representations showing the distribution of barcodes per sample (left), cell type annotations (middle), as well as the number of barcodes per dataset assigned to each cluster (right). **e** Comparison of cellular noise across the main cell populations detected in (**d**). See also Fig. 2e. For better visualization, outliers >0.4 are not included. **f** Differential expression analysis of CD8 naïve T cells (cluster 4 in (**d**)), comparing snRNA-seq versus scRNA-seq samples. Genes were split into ten equally populated bins, based on their mean expression (vertical lines) and genes with no differential expression (red dots) were selected to compare noise levels per gene (see Fig. 2f). Threshold values: fold change (FC) > 1.25, adjusted *P* value (padj) < 0.001. **g** Test of differential noisy genes in CD8 naïve T cells, comparing nuclei versus cell samples. Threshold values: FC > 2, padj < 0.001. **h** Quantification of RNA spots for each cellular compartment from smFISH experiments. See also Fig. 2g,h. Horizontal lines indicate the mean. DC, dendritic cells; NK, natural killer cells, TEM, effector memory T cells; Mono, monocytes.

**
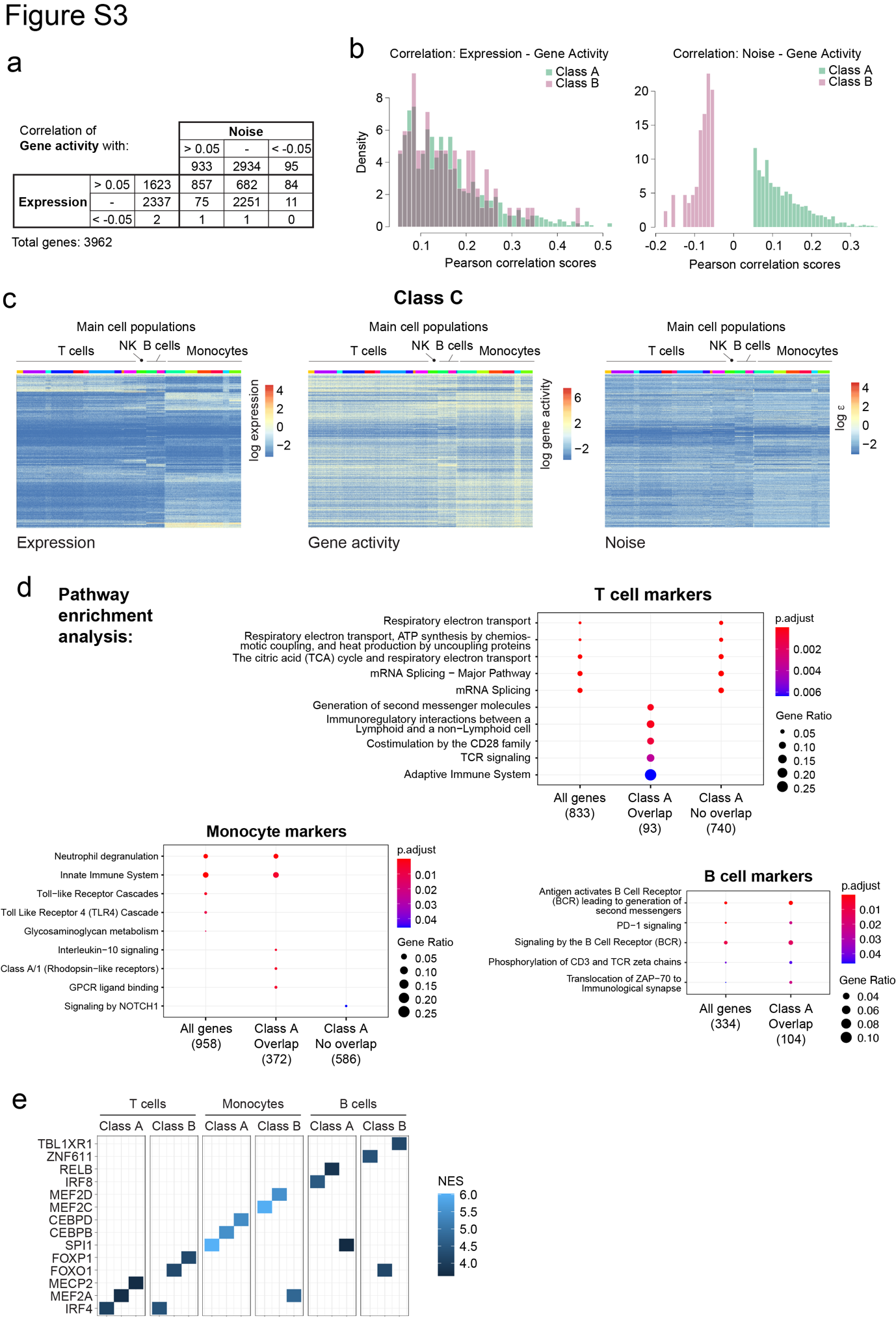
**

**Figure S3. Joint analysis of chromatin accessibility, gene expression, and gene expression noise reveals gene modules with distinct modes of regulation. a** Pearson correlations between gene activity and gene expression, and between gene activity and noise were computed. The contingency table shows the number of genes identified with a positive (> 0.05), negative (< -0.05) or undefined (> -0.05 and < 0.05) correlation. **b** Histograms showing the distribution of Pearson correlation scores of class A and class B genes defined in (**a**) and Fig. 3a-c. **c** Heatmaps showing patterns of expression, gene activity and noise of genes belonging to class C.  **d** Pathway enrichment analysis performed for marker genes of T cells, monocytes and B cells. Marker genes were defined as cell type-enriched genes by performing pairwise differential expression analyses. For each major cell population, marker genes were analyzed in three groups: all genes, marker genes belonging to Class A (“Overlap”), and marker genes not belonging to Class A (“No overlap”). For B cell marker genes, the non-overlapping group was not significantly enriched in any pathway. **e** Motif enrichment analysis performed for genes with increased expression in one of the main cell types, i.e., T cells, monocytes and B cells, that belong to either class A or class B genes. The top three motifs with highest enrichment within each category are shown, and the normalized enrichment score (NES) is indicated.

**
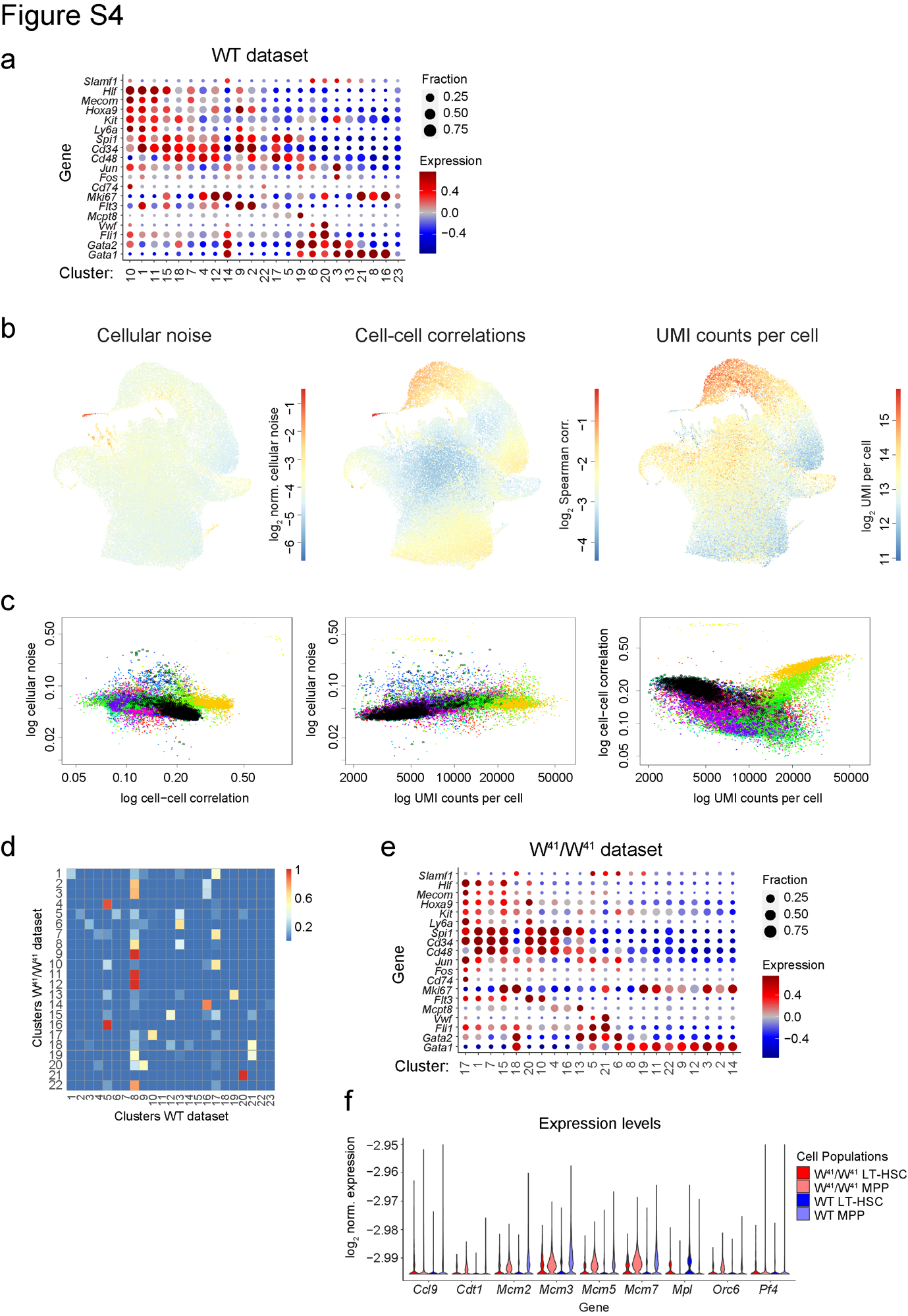
**

**Figure S4. Gene expression noise increases during hematopoietic differentiation. a** Expression of relevant marker genes across cell clusters in the WT dataset of hematopoietic progenitors [34]. Dot size indicates the fraction of cells with positive expression and dot color highlights expression z-score calculated across clusters. Values higher than 0.75 and lower than -0.75 are replaced by 0.75 and -0.75, respectively. See clustering in Fig. 4a. **b** UMAPs highlighting cellular noise (left), local cell-cell correlations (center) and UMI counts per cells (right) across the WT dataset. **c** Scatterplots of pairwise combinations of the quantities displayed in (**b**). Colors correspond to the clusters in Fig. 4a. Data points of the LT-HSCs (cluster 10) are highlighted with a black outline. **d** Heatmap depicting similarity weights of clusters in the W^41^/W^41^ dataset to WT cluster inferred by quadratic programming (“Methods”). **e** As (**a**), but for the W^41^/W^41^ dataset. **f** Violin plot showing normalized expression of genes involved in DNA replication, similar to representation in Fig. 4g. Samples are separated into LT-HSC and the remaining cells, denoted MPP (multipotent progenitors). For better visualization, outliers > -2.95 are not included.

**
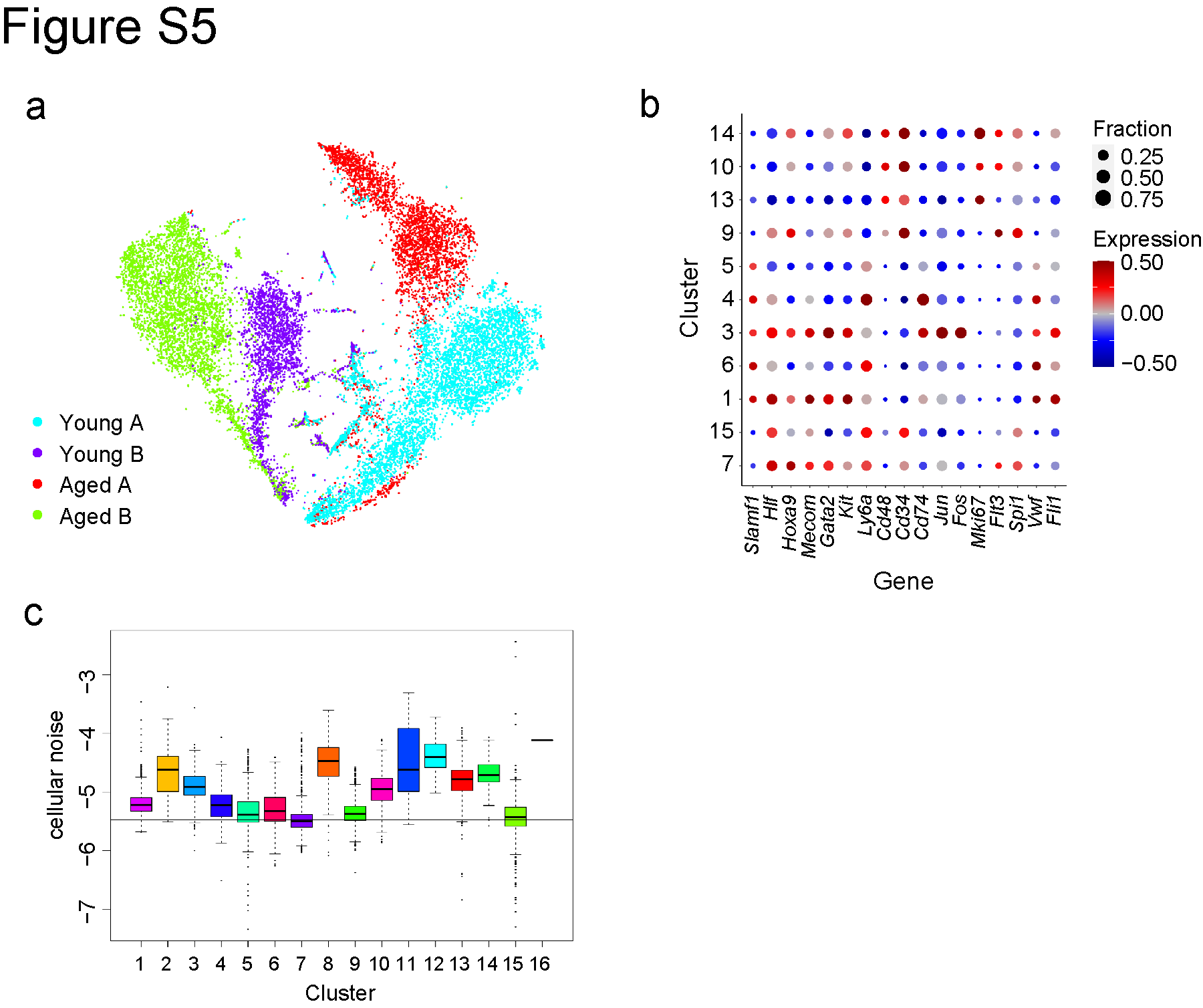
**

**Figure S5.** **Gene expression noise increases in LT-HSCs upon ageing. a** t-SNE map highlighting the four samples processed in two batches from the Hérault et al. (2021) dataset [38]. **b** Expression of relevant marker genes across cell clusters in the dataset. See clustering in Fig. 5a. Dot size indicates the fraction of cells with positive expression and dot color highlights expression z-score calculated across clusters. Values higher than 0.5 and lower than -0.5 are replaced by 0.5 and -0.5, respectively. **c** Quantification of cellular noise across all clusters in the dataset. Horizontal line corresponds to the median noise level of the LT-HSC young A population (cluster 7). Boxes indicate inter-quartile range, and whiskers correspond to ±1.5*IQR of the box limits. Outliers beyond the whisker limits are depicted.

**
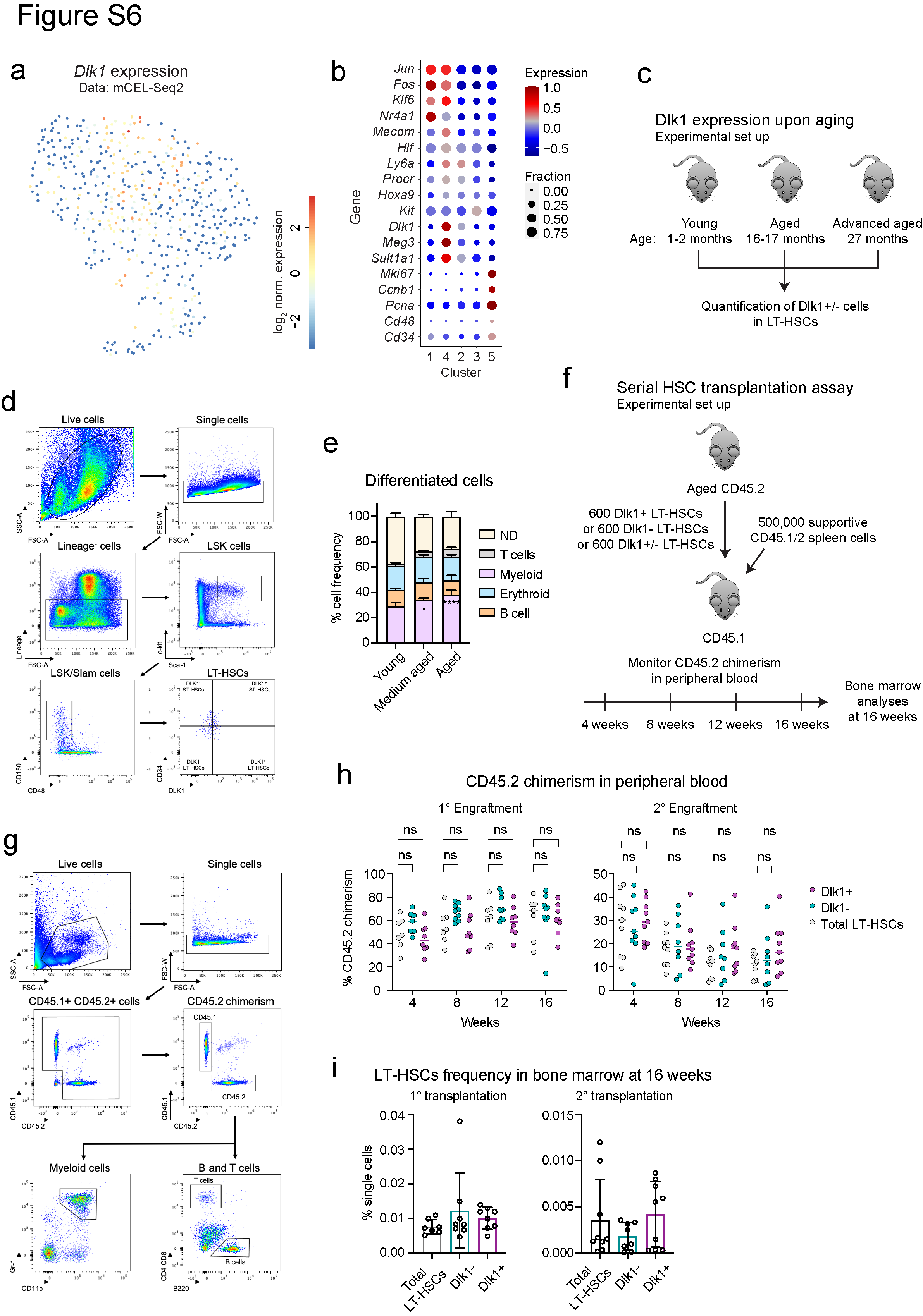
**

**Figure S6. Dlk1 is a marker of quiescence and enhanced self-renewal of aged HSCs. a** UMAP representation highlighting expression of *Dlk1* in the mCEL-Seq2 dataset shown in Fig. 6c,d. **b** Expression of relevant marker genes across cell clusters in the mCEL-Seq2 dataset. Dot size indicates the fraction of cells with positive expression and dot color highlights expression z-score calculated across clusters. Values higher than 1 are replaced by 1. **c** Experimental design for quantification of Dlk1+/- LT-HSC frequency upon aging. **d** Representative gating scheme for sorting Dlk1+/- LT-HSCs, corresponding to the experiment described in (**c**), see “Mehods”. **e** Hematopoietic lineage analysis of bone marrow for the age groups described in (**c**). ND: non-differentiated. Error bars indicate standard deviation. **f** Experimental design of serial HSC transplantation assay, see “Mehods”. **g** Representative gating scheme for monitoring the CD45.2 chimerism, corresponding to the experiment described in (**f**), see “Mehods”. **h** Percentage of CD45.2 chimerism in peripheral blood across the indicated time points for primary (left) and secondary (right) transplantations, corresponding to the experiment described in (**f**), see “Mehods”. **i** Quantification of LT-HSCs in bone marrow 16 weeks post transplantation, showing primary (left) and secondary (right) transplantations. Error bars indicate standard deviation. Barplots and scatterplots: *P* value: ns>0.05, * ≤0.05, ** ≤0.01, *** ≤0.001, **** ≤0.0001 (two-way ANOVA test).

**Table S1. List of public datasets analyzed.**

| Dataset | Reference | Repository / Accession number |
| --- | --- | --- |
| Murine Kit+ hematopoietic progenitor cells from bone marrow | Tusi et al., 2018 [22] | GEO: GSE89754 |
| Human PBMC Single Cell Gene Expression Assay (v3 chemistry) | - | 10x Genomics; https://support.10xgenomics.com/single-cell-gene-expression/datasets/3.0.0/pbmc_10k_v3 |
| Human PBMC, Single Cell Multiome ATAC + Gene Exp. Assay | - | 10x Genomics, https: //support.10xgenomics.com/single-cell-multiome-atac-gex/ datasets/1.0.0/pbmc_granulocyte_sorted_10k |
| Murine hematopoietic progenitor cells from bone marrow | Dahlin et al., 2018 [34] | GEO: GSE107727 |
| Aged and young hematopoietic stem cells from murine bone marrow | Hérault et al., 2021 [38] | GEO: GSE147729 |

**Table S2. List of software packages and tools**

| Sofware Package / Tool | Ussage | Version | Repository / source |
| --- | --- | --- | --- |
| RaceID | Single-cell RNAseq data analysis and VarID2 method | 0.2.6 | https://CRAN.R-project.org/package=RaceID |
| BASiCS | Estimation of gene expression variability | 1.8.1 | https://www.bioconductor.org/packages/release/bioc/html/BASiCS.html |
| Seurat | Single-cell RNAseq data nalysis | 3.9.9 | https://satijalab.org/seurat/articles/install.html |
| Signac | Single-cell ATACseq data nalysis | 1.0.0 | https://stuartlab.org/signac/articles/install.html |
| Harmony | Batch correction | 0.1.1 | https://CRAN.R-project.org/package=harmony |
| RcisTarget | Motif enrichment analysis | 1.16.0 | https://bioconductor.org/packages/release/bioc/html/RcisTarget.html |
| biomaRt | Biological data repository | 2.42.1 | https://bioconductor.org/packages/biomaRt/ |
| BWA | mCELseq2 data aligment | 0.6.2-r126 | https://bio-bwa.sourceforge.net/ |
